# On the Applicability of Ground-Based Microwave Radiometers for Urban Boundary Layer Research

**DOI:** 10.3390/s24072101

**Published:** 2024-03-25

**Authors:** Michael Bartsevich, Kalimur Rahman, Omar Addasi, Prathap Ramamurthy

**Affiliations:** Department of Mechanical Engineering, The City College of New York, New York, NY 10031, USA

**Keywords:** microwave radiometer, urban boundary layer, radiosonde

## Abstract

Significant knowledge gaps exist in our understanding of urban boundary layer processes, particularly the hygrothermal state. The earth system community has successfully used microwave radiometers for several decades. However, the applicability in complex urban environments has never been adequately tested. Here, observations from a microwave radiometer are compared to radiosonde readings in a densely urbanized site in Houston, Texas. The site was influenced by both an urban heat island and the sea breeze phenomenon. The analysis showed significant disagreement between the virtual potential temperature predicted by the microwave radiometer and the radiosonde for all periods within the boundary layer. However, the values were reasonably comparable above the boundary layer. The microwave radiometer incorrectly predicted an inversion layer instead of a mixed layer during convective periods. The microwave radiometer measurements deviated from the radiosonde measurements throughout the lower troposphere for the relative humidity.

## 1. Introduction

One of the most significant knowledge gaps in urban meteorology is the lack of observations of key variables (air temperature, humidity, water vapor, wind) throughout the boundary layer, particularly in the mixed layer [1,2,3]. In recent times, Doppler Lidars and Ceilometers have been used to study the wind characteristics and determine the height of the boundary layer, respectively [4,5,6]; however, measurements of the temperature and humidity have been challenging to obtain. Traditional techniques using radio acoustic sounding systems and radiosondes are often impossible to use due to noise pollution, cost, and security issues [7,8]. Furthermore, the radio acoustic sounding systems cannot reach the deep urban convective boundary layers, and radiosondes need to measure the temperature continuously and are expensive to operate frequently. Microwave radiometers (MWRs) have been widely used to study the tropospheric temperature and humidity profiles; an MWR exploits the absorption and emission frequencies of water vapor and liquid water to determine the hydrophysical state of the atmosphere [9,10]. Herein, we test the applicability of MWRs to urban boundary layer research.

MWRs have been extensively used to study the hydrophysical characteristics of the troposphere. There are dedicated networks around the world that use MWRs for climate studies, weather forecasting, and validation. The European Cloud Observation Network employs 14 radiometers for cloud observations and modeling [11]. The Network for the Detection of Atmospheric Composition Change (NDAC) employs a global network to understand the upper atmospheric concentration of key trace gases [12]. MWRs have been deployed in ship fields (Liu, 1983; Schnitt et al., 2024); both the National Oceanic and Atmospheric Administration and the Department of Energy’s Atmospheric Radiation Measurement program extensively use MWRs [13,14].

While MWRs have been in operation for decades, they have not been adopted for urban climate research. In the last 3 decades, numerous field studies have been conducted in cities worldwide to understand and model the urban environment and the associated climate processes [15,16]. The heterogeneous 3D land cover, with distinct thermal and hygroscopic properties and anthropogenic emissions of heat, water vapor, and pollutants, makes the urban environment very unique compared to other biomes [17]. Due to the increased heat storage and anthropogenic emissions, the thermal boundary layer that develops over cities is very distinct [4,18]. In urban coastal environments, the interaction between the sea breeze and urban heat island leads to unique boundary layer characteristics [19,20]. However, our understanding of these processes is limited due to a lack of heat and humidity measurements throughout the boundary layer. Furthermore, the current models that predict thermal and water transport in the boundary layer have seldom been tested in urban environments. Overall, there is an urgent need to adequately understand the hygrothermal properties of the urban boundary layer.

As reported above, there have been very few studies that have deployed MWRs in urban areas. Ref. [21] validated the MWR with radiosonde data for a mid-latitude urban site in Granada, Spain, and found reasonable biases in both the temperature and humidity profiles and higher biases in the lower troposphere. In Mexico City, [7] compared readings from an MWR, micropulse lidar, and a radiosonde. The study found that the MWR values were in line with the other measurements in predicting the boundary layer height. However, the study fell short of recommending MWRs for temperature measurements. A study in Guangzhou, China, compared four different machine-learning algorithms to see which one yielded the best results for relative humidity (RH) and temperature retrieval when compared to traditional radiosondes. The study found abnormally high root mean square errors for both variables. The primary cause of such a discrepancy was claimed to come from a lack of independent cloud-related information, as well as the influence of solar radiation, especially at low altitudes [22].

The primary aim of this brief note is to evaluate the useability of MWRs in urban boundary layer research. During the Convective-Cloud Urban Boundary-layer Experiment in Houston, Texas, a microwave radiometer was operated continuously from 15 May 2022 to 1 September 2022. During the campaign, nearly 50 radiosondes were launched; most of the launches happened from the same site where the MWR was located. Here, we compare the profiles of the virtual potential temperature and relative humidity observed by both instruments for varying local and synoptic conditions.

## 2. Experimental Setup

**Figure 1:**
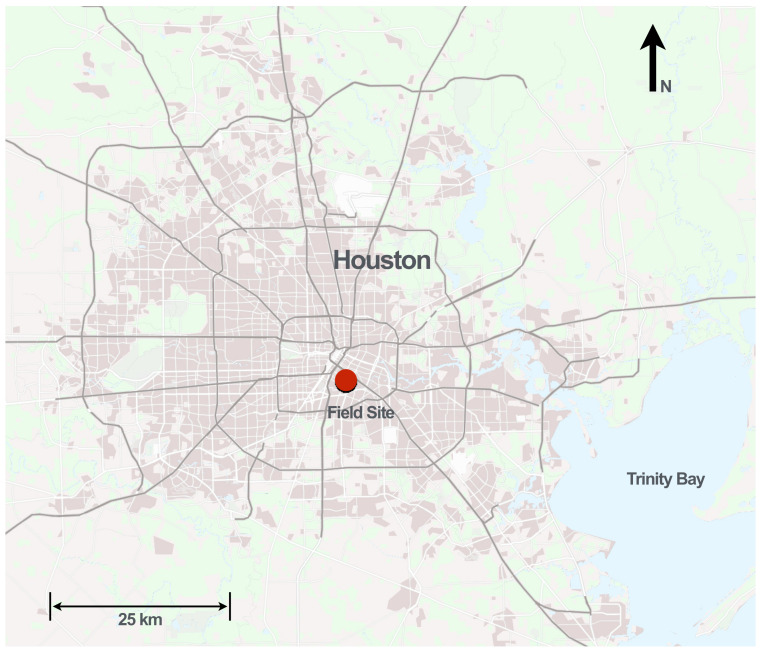
Map showing the location of the field site in Houston, Texas. The highly urbanized location is located just 5 km south of Downtown Houston. The site is located on the campus of the University of Houston.

The data used for this analysis were part of the Convective-Cloud Urban Boundary-layer Experiment (CUBE) campaign organized between 1 June 2022 and 31 August 2022 in Houston, Texas. The field campaign aimed to study the impact of urbanization on convective cloud processes in coastal environments. As part of the study, various ground-based remote sensing and in situ instruments were used to monitor the surface energy budget and atmospheric profiles of the heat and momentum at multiple locations. For the analysis here, we used data from the urban site located on the University of Houston campus. Figure 1 shows the location of the site. The urban site is located within the inner ring road and around 5 km south of Downtown Houston. The site is heavily urbanized, with low vegetative cover; around 85% is covered by built materials (asphalt, concrete, steel, roof). According to the [23] urban landcover classification, it falls under the local climate zone (LCZ) 2. The site is around 35 km west of Trinity Bay and is heavily influenced by the sea/bay breeze.

The urban site in Houston included a flux tower that monitored the surface layer turbulence, heat and mass fluxes, along with a ceilometer and a microwave radiometer; multiple radiosondes were launched from the site. Here, we compare the readings from the MWR to the radiosondes. A Radiometrics^®^ MP-3000A radiometer (RPG Radiometer physics GmbH, Meckenheim, Germany) was used in this study. The theory of operation behind any microwave radiometer profiler is based on the absorption and emission of microwave radiation in the range centered around 22.235 GHz and 59 GHz, which are the absorption frequencies for water vapor and oxygen molecules, respectively [24]. The MWR is a passive instrument that observes the profiles of the temperature, relative humidity, water vapor, and liquid water density. The profiles are estimated using the brightness temperature recorded at several bands: 60 GHz for temperature, 22 GHz for water vapor, and liquid water from bands 22–30 GHz and 51–59 GHz. The mixing ratio of oxygen is invariant with altitude. Hence, the emission at each vertical plane depends on the local temperature; the variation of the emission with the frequency permits radiation from a range of altitudes to reach the instrument, thereby permitting the vertical temperature distribution to be retrieved. After the brightness temperature is collected, it needs to be converted into the profiles of the air temperature and relative humidity. This is performed using neural networks and machine-learning algorithms. The neural networks are trained using data from historical radiosonde soundings collected at or near the site of interest. The network files and the training resources used for the neural network are region-specific and need to be retrained for any additional or new sites.

## 3. Results and Discussion

**Figure 2:**
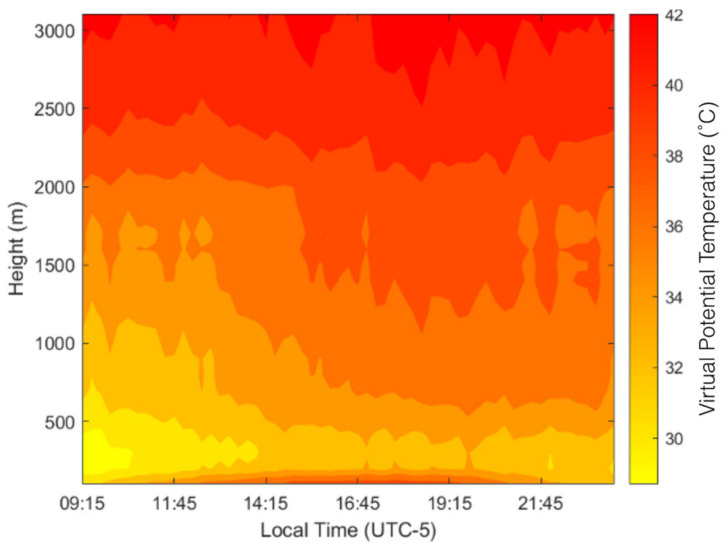
Contour plot showing the daily variability in the VPT in the lower 3000 m for a typical summer day.

Observations from the MWR are compared here with the readings from the radiosonde launches. One of the primary advantages of using an MWR is shown in Figure 2. Here, a 30 min averaged contour plot of the virtual potential temperature (VPT) is shown for 19 June 2022. We have restricted our analysis to the lower 3000 m; however, the MWR is capable of retrieving data from up to 10,000 m. The sample plot shows the continuous variability in the VPT throughout the day for the entire boundary layer. Here, the VPT values decrease with the height near the surface, exhibiting a super-adiabatic layer, and then increase with the height. During the convective period, the VPT values range between 33 and 39 °C in the lower 500 m, with higher values close to the surface. The values decline and increase again in the levels between 500 and 3000 m. The radiometer can also sense the variability in the water vapor and liquid water in the atmosphere. The observations from the radiosondes, on the other hand, are not continuous; on most days, 1–2 sondes were launched during the convective period. We deployed the WindSonde radiosondes that are more suitable for boundary layer observations; the radiosondes require less voluminous mylar balloons and traverse the atmosphere with lower velocity and a higher data retrieval rate. The WindSonde radiosondes were specifically developed for boundary layer observations, while traditional radiosondes primarily focus on the middle and upper troposphere.

The launches were classified based on the time of the day (daytime and night-time) and the wind characteristics (sea breeze and land breeze); daytime here refers to convectively active periods between 09:00 and 18:00 local time, while all the other launches were labeled as night-time. The radiosonde wind direction data from 0 to 3000 m was used as the criteria to classify the data as sea breeze or land breeze periods; periods when the winds were from 90–200 degrees were considered as a sea breeze, and the rest were classified as a land breeze.

**Figure 3:**
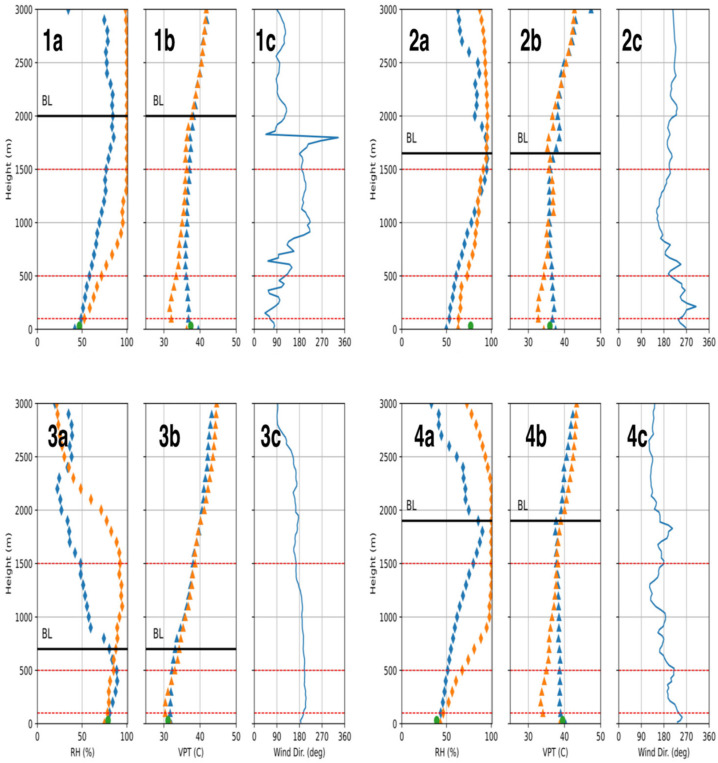
Panel plots comparing the microwave radiometer (MWR) measurements to the radiosonde (RDS) measurements. The RH here refers to the relative humidity, and the VPT refers to the virtual potential temperature. Panel 1 (06/22/2022 at 13:02 Houston time) compares the observations for a typical sea breeze daytime period; panel 2 (08/01/2022 at 15:30 Houston time), for a land breeze daytime period; panel 3 (07/20/2022 at 23:43 Houston time), for a sea breeze night-time period; panel 4 (07/21/2022 at 13:59 Houston time), for a sea breeze daytime period with heatwave conditions. The green dot corresponds to the ground condition recorded by an in situ monitor.

Figure 3 compares the radiosonde observations to the MWR readings for four different days. The panel plots compare the relative humidity (RH) and VPT profiles retrieved from the MWR and radiosondes for a daytime sea breeze (1a, 1b, 1c), daytime land breeze (2a, 2b, 2c), night-time sea breeze (3a, 3b, 3c) and daytime sea breeze heatwave conditions (4a, 4b, 4c). While the panel plots compare a single launch episode, they represent their typical periods. Table 1 below shows the average difference between the MWR and radiosonde observations for the entire study period. The panels are subdivided into the surface layer (lower 100 m), lower mixed layer (100–500 m), and upper mixed layer (500–1500 m). Furthermore, the respective boundary layer heights are also marked.

From all the panel plots, it is clear that the MWR values do not agree with the radiosonde values; the difference is very high in the lower 500 m for the VPT, and for the relative humidity, the values are much farther apart for up to 1500 m.

During the daytime sea breeze case (1a and 1b), the VPT values from the radiosondes show a classical convective boundary layer; there are high VPT values close to the surface around 37 °C, which then decrease in the surface layer; above 500 m, the values are uniform until the cloud top layer. Above the boundary layer, the VPT values increase with the height. The MWR shows increasing VPT values in the lower 500 m; it significantly underpredicts the VPT, and an almost 7 °C difference between the MWR and radiosonde values is observed around 200 m above ground level. The VPT values increase with the height, dynamically showing a deep inversion layer. Above the boundary layer, the VPT values are in strong agreement with the radiosonde values. The RH values show considerable disagreement throughout the boundary layer. The MWR predicts a highly saturated boundary layer, with values close to 100% above 1000 m; however, the radiosondes show increasing RH values, with a maximum of around 80% at 2000 m. The radiosondes show decreasing RH values above the boundary layer, while the MWR observations show a completely saturated atmosphere.

For the daytime land breeze case (2a and 2b), the MWR predicts a steep lapse rate and inversion layer in the lower 700 m. The radiosonde values initially increase and remain uniform until 1700 m (depth of the boundary layer). Above 2000 m, there is good agreement. For the RH, the MWR overpredicts by at least 10% within the boundary layer. However, the profile pattern seems consistent with the radiosonde readings. Unlike the VPT, above 2000 m, there is little agreement in the RH values.

For the night-time case (3a and 3b), while the difference between the VPT values observed by both instruments is much smaller, the dynamics are still different. The radiosonde values show a low lapse rate and a shallow mixed layer. However, the MWR shows a strong inversion layer, with the VPT values increasing from 30 °C to 35 °C between 200 and 700 m. In comparison, the radiosondes show constant VPT values between these levels. Similar to the other two episodes discussed above, the VPT values from both instruments are similar above the boundary layer. The RH values observed during the night-time have little to no agreement with the values observed by the radiosondes, with differences as high as 50%.

Houston experienced heatwave conditions between 16 and 21 July 2022. A record warm low temperature of 27 °C was set on July 20th. Panel plots 4a and 4b are from July 21st around 14:00 local time. The average near-surface air temperature was above 37 °C. This amplification is visible in the radiosonde reading; the near-surface VPT was one of the highest recorded, around 40 °C, nearly 4–5 °C warmer than the other two days shown in plots 1b and 2b. The radiosonde data show the high VPT throughout the boundary layer, and the lapse rate is lower compared to the other two days. The VPT values hardly change within the boundary layer due to the high-pressure system. The MWR readings are significantly different. The highest differences are observed around 100 m above the ground level, where the MWR values are 6 °C lower than the radiosonde readings. Similar to the above two cases (1b and 2b), the MWR values show an inversion layer, with the VPT values increasing throughout the boundary layer. Unlike the other two daytime episodes, the VPT values differ even above the boundary layer. The RH values observed by the MWR are significantly different compared to the radiosonde readings; the differences are comparable to the sea breeze daytime case shown in the plot.

The analysis above is consistent across all the days; Table 1 shows the average mean bias error for the different levels for both the VPT and RH for the land breeze and sea breeze days. All the data here correspond to convectively active periods. The VPT is underpredicted throughout the lower atmosphere, with maximum variability in the lower 500 m. On average, the values observed by the MWR were more than 4 °C less than those observed by the radiosondes for the sea breeze and land breeze periods. The disagreements were lower for heights above 500 m, between 1 °C and 1.7 °C. For the RH, the mean bias error averaged between 35% and 3.8%, with low MBEs for heights below 500 m and high errors above the boundary layer.

Overall, the analysis has shown that the MWR fails to observe the hygrothermal state of the urban boundary layer accurately. The MWR underpredicts the virtual potential temperature within the boundary layer and highly overpredicts the RH throughout the lower atmosphere. In addition to the observed discrepancy in the VPT values, the MWR erroneously shows an inversion layer during the convective period. However, above the boundary layer, the MWR does a reasonable job of estimating the VPT.

While it is beyond the scope of this article to examine the reason behind MWRs’ poor performance, we find the significant disagreement in the boundary layer temperature and humidity measurements to be related to three distinct factors: the presence of aerosols in the urban environment, the MWR training, and the neural network algorithm. Coastal urban environments are highly challenging due to the high convective activity and the presence of natural and industrial aerosols. During the summer months, Houston is heavily influenced by convective activity, with the presence of large cumulonimbus clouds, which can potentially scatter the radiation, thereby limiting the accuracy of the MWR observations. Ref. [21] observed higher biases on cloudy days compared to clear days at a continental site.

The algorithms used could be improved using new AI/ML techniques. Ref. [14] found a high degree of variability between different AI/ML algorithms. They tested DL, gradient boosting, extreme gradient boosting, and random forest methods and found deep learning to be better suited; hence, there is some potential for improvement here.

Finally, MWRs need to be trained using local datasets; in the US, historical radiosonde launches are used for the training. However, few launches take place in urban environments. The majority of the radiosonde launches that are used to train the MWRs happen in rural and ex-urban environments. Hence, the current historical dataset might not represent the complex urban boundary layer processes.

## 4. Conclusions

This article reports on the useability of microwave radiometers for urban boundary layer research. Temperature and humidity values from the MWR were compared to radiosondes for multiple local conditions in Houston, Texas. Overall, during most periods, the MWR underperformed; the virtual potential temperature values were much lower in the boundary layer, and the RH values were high. For the VPT, the values above the urban boundary layer were reasonable; however, for the RH, the accuracy of the values remained poor throughout the lower atmosphere. Worryingly, the MWR predicts an inversion layer instead of a mixed layer typically observed during convective periods.

The error observed could potentially be due to the presence of excess cloud scatter or issues with the training methodology used to determine the temperature and moisture profiles. Moreover, our experiment was conducted in a coastal city that experiences high convective activity during the summer months. Houston is a highly industrialized city with high anthropogenic aerosol concentrations in the lower atmosphere. The results could very well be different in another environment.

## Figures and Tables

**Table 1:** Average mean bias (MBE) error for section 1 (0–500 m), section 2 (500–1500 m) and section 3 (1500–3000 m) for sea breeze and land breeze conditions during daytime for the (**a**) relative humidity (%) and (**b**) virtual potential temperature (°C). (The mean bias error took the MWR and subtracted the radiosonde to create determine if the MWR was overpredicting or underpredicting).

(a) Relative Humidity (%)			
	S1 (0–500 m)	S2 (500–1500 m)	S3 (1500–3000 m)
**Sea breeze**	3.860	20.576	35.076
**Land breeze**	8.775	15.883	30.217
**(b) Virtual Potential Temperature (°C)**			
	**S1 (0–500 m)**	**S2 (500–1500 m)**	**S3 (1500–3000 m)**
**Sea breeze**	−4.144	−1.702	−1.092
**Land breeze**	−4.490	−1.307	−1.547

## Data Availability

No new data were created or analyzed in this study. Data sharing is not applicable to this article.
